# Diagnostic accuracy of semiquantitative point of care urine albumin to creatinine ratio and urine dipstick analysis in a primary care resource limited setting in South Africa

**DOI:** 10.1186/s12882-021-02290-5

**Published:** 2021-03-20

**Authors:** Sean D. Currin, Mwawi S. Gondwe, Nokthula B. Mayindi, Shingirai Chipungu, Bongekile L. Khoza, Stephen Tollman, June Fabian, Jaya A. George, On behalf of the ARK Consortium

**Affiliations:** 1 Department of Chemical Pathology, Faculty of Health Sciences, University of Witwatersrand, 7 York Road, Parktown, Johannesburg, 2193 South Africa; 2 National Health Laboratory Service, Johannesburg, South Africa; 3 Faculty of Health Sciences, Medical Research Council/Wits University Rural Public Health and Health Transitions Research Unit (Agincourt), School of Public Health, University of Witwatersrand, Johannesburg, South Africa; 4 International Network for the Demographic Evaluation of Populations and their Health (INDEPTH) Network, Accra, Ghana; 5 Wits Donald Gordon Medical Centre, Faculty of Health Sciences, School of Clinical Medicine, University of Witwatersrand, Johannesburg, South Africa

**Keywords:** Point of care, Urine albumin creatinine ratio, Dipstick, Chronic kidney disease

## Abstract

**Background:**

The prevalence of chronic kidney disease (CKD) is predicted to rise over the next few decades. In resource-limited settings access to central laboratory services is limited. Point-of-care (POC) urine dipstick testing offers the potential to detect markers of kidney damage (albuminuria) as well as markers of other disease processes. We evaluated the diagnostic accuracy of the semi-quantitative albumin-creatinine ratio (ACR) Sysmex UC-1000 POC urine dipstick system as well as the extent of other abnormal dipstick findings in urine.

**Methods:**

700 participants from a rural area in South Africa were screened for albuminuria. A spot urine sample was used to measure POC and central laboratory ACR. We determined the sensitivity, specificity, positive predictive value and negative predictive value of the POC ACR, and recorded dipstick parameters.

**Results:**

The prevalence of albuminuria was 11.6% (95%CI; 9.3–14.2). Those with albuminuria had higher mean diastolic (82 vs 79 mmHg, *p* = 0.019) and systolic (133 vs 128 mmHg, *p* = 0.002) blood pressures and a higher proportion of diabetes mellitus (17.6 vs 4.9%, *p* < 0.001). The sensitivity of the POC ACR system was 0.79, specificity 0.84, positive predictive value 0.39 and negative predictive value 0.97. The sensitivity improved to 0.80, 0.85, 0.85 and 0.89 in those with elevated blood pressure, diabetes mellitus, HIV positive status, and those 65 years and older, respectively. Abnormalities other than albuminuria were detected in 240 (34.3%) of the samples; 88 (12.6%) were positive for haematuria, 113 (16.1%) for leucocytes, 66 (9.4%) for nitrites and 27 (3.9%) for glycosuria.

**Conclusion:**

Our study shows that POC ACR has good negative predictive value and could be used to rule out albuminuria when screening for CKD. Additionally, a high proportion of participants had other urine abnormalities detected with dipsticks which may reflect kidney disease or co-morbid untreated genitourinary pathology such as urinary tract infections or endemic schistosomiasis with important implications for CKD.

**Supplementary Information:**

The online version contains supplementary material available at 10.1186/s12882-021-02290-5.

## Background

Sub-Saharan Africa (SSA) has a high burden of infectious diseases (ID), mostly from HIV and tuberculosis, and an emerging burden of non-communicable diseases (NCD) [1, 2]. Together, ID and NCD are risk factors for chronic kidney disease (CKD), which has a prevalence of 10.7% in SSA [3]. The prevalence of CKD is predicted to rise disproportionately in low- and middle-income countries (LMIC) [4]. Therefore, early detection of CKD and appropriate management of risk factors for progression is an essential public health priority.

Persistent albuminuria is one criterion for the diagnosis of CKD, and an independent risk factor for adverse kidney and cardiovascular outcomes [5–9]. Albuminuria, measured on more than one occasion, as a spot urine albumin to creatinine ratio (ACR), is now included as one of the diagnostic criteria for CKD [10]. Screening for albuminuria is recommended in certain high-risk groups such as those with diabetes mellitus, however results should be confirmed by a central laboratory [11, 12].

The lack of access to central laboratory services in LMICs is a major hurdle obstructing widespread implementation of CKD screening programs. While limited laboratory access is acknowledged in the 2012 KDIGO guidelines, there are no recommendations for using point of care (POC) devices in resource-limited settings (RLS) [10]. POC testing provides actionable results in real-time for clinical decision-making [13]. In South Africa (SA), the efficacy of POC testing is well established for diagnosing and managing patients with HIV and tuberculosis [14]. Using the existing framework established for POC testing with ID, we propose that POC testing for screening and early detection of CKD is feasible in our setting. Previous studies [15–22] have shown good negative predictive values with fair sensitivity for semi-quantitative ACR POC testing, which suggests utility as a screening test for CKD. Additionally, the ability of urine dipsticks to detect other disease markers besides albuminuria offers added benefit.

The aim of our study was to evaluate the diagnostic accuracy of a POC semi-quantitative ACR system in a RLS in rural SA, and evaluate additional urinary abnormalities discovered on dipstick analysis.

## Methods

### Study design

**Fig. 1:**
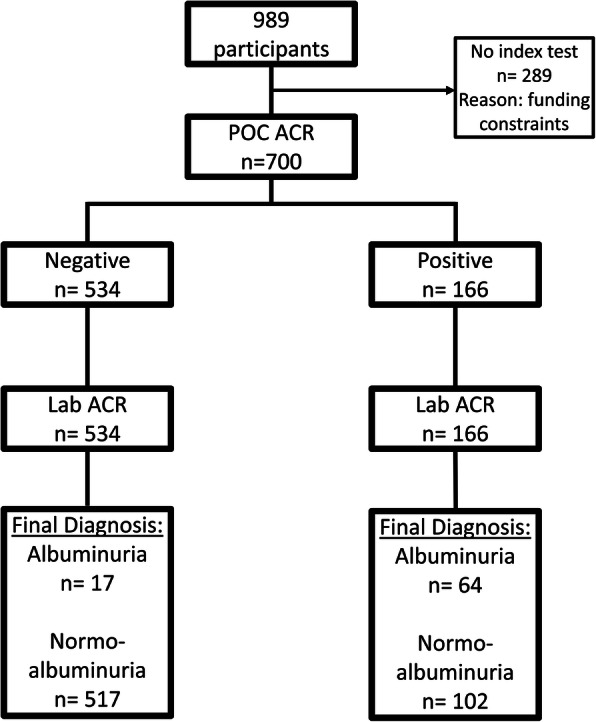
Flow of participants through the study. POC ACR is the index method while Lab ACR is the reference method

Our sub-study comprised part of the prospective African Research on Kidney (ARK) study, the methods of which have been published [23]. To summarise, the ARK study was conducted in the Agincourt Health and Demographic Surveillance Site, Mpumalanga province, SA. There were two phases of the ARK study; the first phase (November 2017 – October 2018) screened a population-based sample of 2020 rural Africans for kidney disease and associated risk factors, those between the ages of 20 and 80 were considered eligible; the second phase (November 2018 – July 2019) comprised a subsample of the first phase, stratified by CKD stage. (Fig. 1).

In phase one of the ARK study, a CKD-risk questionnaire was administered, blood pressure (BP) was measured (as per the Joint National Committee on Prevention, Detection, Evaluation, and Treatment of High Blood Pressure guidelines [24]), and voluntary counselling and HIV testing was offered to all participants according to the SA Department of Health Guidelines [25]. In the field, POC tests were performed including urine dipstick analysis using Roche (Mannheim, Germany) Combur 10® test strips on fresh spot urine samples.

The second phase of the ARK study recruited 989 participants and included the same BP and HIV testing protocols as the first phase. Spot urine samples were refrigerated overnight (2 - 8 °C) and processed the following morning for storage at -80 °C. Urine samples were transported at -80 °C from the study site to a central laboratory where they were processed over a nine-day period. Each day, 81 samples were separated into two aliquots, one aliquot was tested using central laboratory ACR, the other aliquot was tested using a POC semi-quantitative dipstick method. Central laboratory ACR results were not known while POC ACR was performed and vice versa. 700 urine samples out of the 989 where selected based on the order in which they were sent to the central laboratory, all 700 samples were processed. Funding constraints limited the sample size to 700 out of 989.

### POC ACR and dipstick analysis

Urine dipstick analysis was performed using Sysmex Corporation (Kobe, Japan) 12S® urine dipsticks together with the Sysmex UC-1000® semi-automated urine chemistry analyser. The 12S dipsticks measure the following parameters: urobilinogen, red blood cells, haemoglobin, protein, glucose, ketones, bilirubin, nitrites, specific gravity, leukocytes, pH, creatinine, and albumin with colorimetric dipstick assays. The Sysmex UC-1000® system optically reads reaction results from 12S dipsticks using multi-wavelength reflectance photometry thereby providing semi-quantitative results. The semi-automated reading of strips eliminates inter-individual variation in interpreting dipstick results. The albumin concentration is measured as 10, 30, 80, or 150 mg/L using the protein error of pH indicator method, while the creatinine concentration is measured as 10, 50, 100, 200, or 300 mg/dL using the Benedict-Behre method; thereafter the ACR is calculated as dilute (albumin 10 mg/L and creatinine 10 mg/dL), normal (< 30 mg/g), 1+ (30–300 mg/g), or 2+ (> 300 mg/g). Internal quality control was performed each day before any samples were run.

### Measurement of laboratory ACR

Laboratory albumin and creatinine measurements were performed on the Cobas c502 module® (Roche Diagnostics, Mannheim), with urine albumin (mg/L) determined using an immunoturbidimetric assay while urine creatinine (mg/dL) was determined using the kinetic Jaffe method. This was considered an appropriate reference method due to widespread usage in central laboratories in South Africa. During the nine days of measurements a single lot was used for both urine albumin and creatinine assays, internal quality control was performed twice daily as per standard practices within the central laboratory, together with an external quality assurance program.

### Statistical analysis

Laboratory measurement of ACR was used as the reference method to which POC ACR was compared. Laboratory ACR was considered positive if ≥30 mg/g and negative if < 30 mg/g. POC ACR was considered positive if falling in the “1+” or “2+” (≥30 mg/g) groups whilst negative if falling in the normal group (< 30 mg/g). Samples in which the laboratory albumin was < 3 mg/L (limit of quantification) were considered as negative, as were POC ACR samples which were dilute (albumin < 10 mg/L and creatinine < 10 mg/dL). All 700 samples were included for analysis.

Statistical analysis was performed using Tibco Statistica 13® and MedCalc 19.1.3®. Continuous data were compared using the students T-test and Mann-Whitney test when appropriate, while categorical data were compared using *χ* ^2^ test. The prevalence of albuminuria, sensitivity, specificity, positive (PPV) and negative predictive values (NPV), and likelihood ratios of POC testing were calculated using cross-tabulation. 95% confidence intervals were calculated according to the exact Clopper-Pearson method for sensitivity and specificity, according to the Miettinen-Nurminen method for likelihood ratios and according to the Mercaldo-Wald method for predictive values. A *p*-value < 0.05 was considered statistically significant.

## Results

### Study population

We analysed 700 urine samples; participant demographics and clinical characteristics are listed in Table 1. Mean systolic and diastolic BP were significantly higher amongst those with laboratory albuminuria, as was a history of diabetes mellitus; however, a history of hypertension was not. The prevalence of albuminuria was 11.6% (95%CI; 9.3–14.2), based on the central laboratory ACR measurements (≥30 mg/g).

**Table 1:** Demographic and clinical features

	Laboratory ACR (mg/g)			p^c^
	< 30	≥30	> 300	
Number of patients	619	81	9	
Age (years)	45 (25)^a^	43 (23)^a^	55 (25)^a^	0.680^d^
BMI (kg/m^2^)	28.1 (6.2)	27.3 (6.5)	27.0 (7.0)^a^	0.239^e^
Systolic BP (mmHg)	128 (14.9)	133 (18.1)	144 (24.0)^a^	0.002^e^
Diastolic BP (mmHg)	79 (8.6)	82 (9.9)	83 (7.0)^a^	0.019^e^
Male (%)^b^	211 (34.1)	31 (38.3)	4 (44.4)	0.457^f^
Hypertension (%)^b^	154 (26.0)	25 (33.8)	3 (37.5)	0.153^f^
Diabetes Mellitus (%)^b^	29 (4.9)	13 (17.6)	2 (25.0)	< 0.001^f^
Smoker (%)^b^	85 (14.3)	11 (14.9)	0 (0)	0.902^f^
HIV reactive (%)^b^	119 (20.1)	20 (27.0)	1 (12.5)	0.246^f^

Data presented as mean (SD) or n (%) unless otherwise indicated

^a^Data presented as median (IQR)

^b^Missing values for all patients = 33

^c^Comparison between laboratory negative (< 30 mg/g) and laboratory positive (≥30 mg/g) groups

^d^Mann-Whitney test

^e^Students T-test

^f^ *χ* ^2^ test

Hypertension, diabetes mellitus, smoker status obtained from medical questionnaire

HIV status obtained from medical questionnaire. If previously tested, participants were asked their status; those who did not know their status, or previously tested negative, were offered voluntary counselling and testing during the home screening (see methods)

Measured blood pressure (BP): see methods

### Clinical performance of the POC ACR

The central laboratory method identified 81 participants as having albuminuria (≥ 30 mg/g) whilst the POC method, performed on the same day, identified 166 participants with albuminuria. No adverse events were reported during testing or collection. In total the POC reader misclassified 119 samples (17%) with 102 false positives, and 17 false negatives. Sensitivity of the POC ACR to detect albuminuria (ACR ≥ 30 mg/g) was 0.790 (95%CI; 0.689–0.865), and specificity was 0.835 (95%CI; 0.804–0.862). A positive likelihood ratio of 4.80 (95%CI; 3.86–5.89) and negative likelihood ratio of 0.25 (95%CI; 0.16–0.37) was obtained for POC ACR. Predictive values of 0.968 (95%CI; 0.952–0.979) for negative results and 0.386 (95%CI; 0.337–0.436) for positive results were obtained. Sensitivity and specificity to predict ACR > 300 mg/g was 1.00 (95%CI;0.664–1.00) and 0.987 (95%CI;0.975–0.994) respectively; with predictive values of 1.00 (95%CI; 0.995–1.00) for negative results and 0.50 (95%CI;0.343–0.657) for positive results.

The sensitivity and specificity of the POC ACR system varied among different groups of participants. There was reduced sensitivity (76%) in those with a history of hypertension (medical questionnaire), however those with raised blood pressure [26] showed an improved sensitivity and specificity of 80 and 86% respectively. The same is true of those with a history of diabetes mellitus, with an improved sensitivity of 85%, however, specificity was lower (66%) in this group. Smokers, those with HIV, and those 65 years or older showed improved sensitivity. PPV remained low in all groups ranging from 0.26 to 0.52, while NPV was high ranging from 0.90 to 0.99. (Table 2).

**Table 2:** Classification of albuminuria as positive or negative by POC and laboratory ACR

All patients (700)			Sensitivity	0,79
	Positive POC (*n* = 166)	Negative POC (*n* = 534)	Specificity	0,84
Positive lab ACR (*n* = 81)	64	17	PPV	0,39
Negative lab ACR (*n* = 619)	102	517	NPV	0,97
**Hypertension (179)**			Sensitivity	0,76
	Positive POC (*n* = 47)	Negative POC (*n* = 132)	Specificity	0,82
Positive lab ACR (*n* = 25)	19	6	PPV	0,40
Negative lab ACR (*n* = 154)	28	126	NPV	0,95
**DM (42)**			Sensitivity	0,85
	Positive POC (*n* = 21)	Negative POC (n = 21)	Specificity	0,66
Positive lab ACR (n = 13)	11	2	PPV	0,52
Negative lab ACR (*n* = 29)	10	19	NPV	0,90
**Smoker (96)**			Sensitivity	0,82
	Positive POC (*n* = 26)	Negative POC (*n* = 70)	Specificity	0,80
Positive lab ACR (*n* = 11)	9	2	PPV	0,35
Negative lab ACR (*n* = 85)	17	68	NPV	0,97
**HIV (139)**			Sensitivity	0,85
	Positive POC (*n* = 41)	Negative POC (*n* = 98)	Specificity	0,80
Positive lab ACR (*n* = 20)	17	3	PPV	0,41
Negative lab ACR (*n* = 119)	24	95	NPV	0,97
**BP: Diastolic ≥ 80 OR Systolic ≥ 130 (389)**			Sensitivity	0,80
	Positive POC (*n* = 93)	Negative POC (*n* = 296)	Specificity	0,86
Positive lab ACR (*n* = 56)	45	11	PPV	0,48
Negative lab ACR (*n* = 333)	48	285	NPV	0,96
**Age ≥ 65 (105)**			Sensitivity	0,89
	Positive POC (*n* = 31)	Negative POC (*n* = 74)	Specificity	0,76
Positive lab ACR (n = 9)	8	1	PPV	0,26
Negative lab ACR (*n* = 96)	23	73	NPV	0,99

Hypertension, diabetes mellitus (DM), smoker status obtained from medical questionnaire

HIV status obtained from medical questionnaire. If previously tested, participants were asked their status; those who did not know their status, or previously tested negative, were offered voluntary counselling and testing during the home screening (see methods)

Measured blood pressure (BP): see methods

PPV: positive predictive value

NPV: negative predictive value

**Fig. 2:**
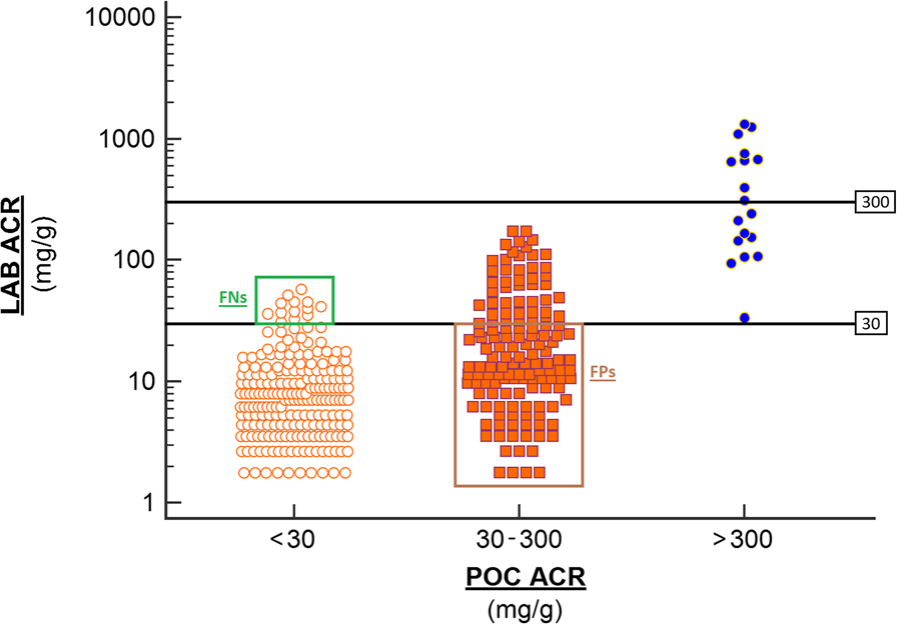
Laboratory ACR (reference) versus POC ACR (index). The horizontal lines represent 30 mg/g and 300 mg/g. 30 mg/g represents the threshold for albuminuria while 300 mg/g is the threshold for 2+ albuminuria (as defined by the POC system). FNs represent false negative results by the POC method while FPs represent false positive results by the POC method

False negative values ranged from 30.9–57.5 mg/g, and those for false positives ranged from 1.8–29.2 mg/g. All nine samples with laboratory ACR > 300 mg/g were correctly identified as “2+” (> 300 mg/g) according to POC ACR, a further nine samples were classified as > 300 mg/g while laboratory ACR classified them as albuminuria (> 30 mg/g) but less than 300 mg/g. POC ACR correctly classified 572 (82%, 95%CI;79–85) of the samples into the correct KDIGO albuminuria stages of A1 (< 30 mg/g), A2(30 – 300 mg/g) and A3 (> 300 mg/g). Accuracy was 97% (95%CI;95–98), 31% (95%CI;24–39), and 50% (95%CI;26–74) in groups A1, A2 and A3 according to POC ACR. (Fig. 2).

### Additional urine dipstick abnormalities

240 (34.3%) of the 700 samples had abnormal findings other than ACR, protein to creatinine ratio, pH, or specific gravity. 65% (53/81) of laboratory confirmed albuminuria samples had additional abnormalities on urine dipstick. While 30% (187/619) of the samples without laboratory confirmed albuminuria demonstrated additional urinary dipstick abnormalities. Using the dipstick method 88 (12.6%) participants had haematuria including 50 in the laboratory negative group, and 113 (16.1%) had leukocyturia.

Urine dipstick findings were compared between those with laboratory positive and negative ACR. Haematuria and glycosuria were significantly higher in the laboratory positive compared to the laboratory negative group (46.9% vs 8.1% and 11.1% vs 2.9% respectively). All abnormalities were seen with a higher proportion in those with laboratory positive ACR except for leucocytes, although not statistically significant. (Table 3).

**Table 3:** Other dipstick findings besides ACR in phase 1 and phase 2

	Blood^e^	Urobilinogen	Bilirubin	Glucose	Leucocytes	Nitrites
**Phase 2 (*n*** **= 700) (Sysmex 12S**® **strips)**^**a**^						
Lab ACR ≥30 (n and %)	38 (46.9)	1 (1.2)	2 (2.5)	9 (11.1)	12 (14.8)	12 (14.8)
Lab ACR < 30 (n and %)	50 (8.1)	2 (0.3)	4 (0.6)	18 (2.9)	101 (16.3)	54 (8.7)
TOTAL (%)	88 (12.6)	3 (0.4)	6 (0.9)	27 (3.9)	113 (16.1)	66 (9.4)
p^d^	< 0.001	0.238	0.094	< 0.001	0.730	0.078
**Phase 1 (*n*** **= 1993)**^**c**^ **(Roche Combur 10**® **strips)**^**b**^						
TOTAL (%)	649 (32.6)	36 (1.8)	46 (2.3)	64 (3.2)	603 (30.3)	49 (2.5)

All data n (%)

^a^Performed on urine after a freeze thaw cycle, semi-quantitative analysis performed with Sysmex UC-1000® device (see methods)

^b^Performed on fresh urine during phase one, semi-quantitative analysis performed with visual inspection (see methods)

^c^Missing data for 27 (2 refused, 25 missing data)

^d^ *χ* ^2^ test Lab ACR ≥30 vs Lab ACR < 30 (all phase 2)

^e^Haemoglobin or erythrocytes detected by dipstick method

### Effect of freezing on semi-quantitative POC dipstick parameters

Freezing had a minimal effect on dipstick parameters with a small increase in false negatives and no increase in false positives being seen for haematuria and leukocyturia. (See Supplementary for detailed results and methodology).

## Discussion

Our study represents the largest evaluation of a POC ACR compared to laboratory ACR to date. Similar to previous studies [15–22] we found that the POC analyser has good rule out utility for albuminuria, as indicated by the NPV. Our study differs from previous studies in that we simultaneously evaluated additional urine dipstick parameters aside from albuminuria. Similar studies [15–21] have looked at semi-quantitative POC ACR from different manufacturers, however this is the first published study on the Sysmex UC-1000® instrument. The fully automated Sysmex UC-3500® has previously been validated showing excellent precision and good analytical accuracy [22].

When using ACR to screen for albuminuria, the recommended sample is an early morning urine sample. Previous studies have used random urine [15, 18, 20] or early morning samples [16, 17, 19, 21] with sensitivity of random urine samples being 0.83 [15], 0.79 [18], and 0.63 [20] while the sensitivity of early morning samples was 0.90 [16], 0.90 [17], 0.75 [19], 0.92 [21] and 0.80 [21], suggesting a possible advantage of early morning sampling [27].

Our findings show the POC device would be useful for excluding albuminuria, but any positive result would need to be confirmed with a laboratory ACR. This in keeping with other studies where NPV ranged from 0.89–0.99 [15, 16, 18, 19, 21, 22] with one study reporting a NPV of 0.71 [17]; while positive predictive value was low for all studies ranging from 0.46–0.82 [15–19, 21, 22]. Similarly, only one study [16] reported a positive likelihood ratio above 10 indicating utility in ruling in disease.

The improved sensitivity seen in participant groups with known risk factors for CKD could help guide screening strategies. The discordance in sensitivities between a history of hypertension (medical questionnaire) and raised BP during examination could point to the effect of antihypertensives, particularly angiotensin-converting enzyme inhibitors, on albuminuria [28]. Screening strategies targeted at those with a history of diabetes mellitus, the elderly, those who present with elevated blood pressure, and those with HIV are justified, and in keeping with management guidelines [29, 30]. Our findings corroborate those of previous studies: in the study by Graziani et al. [16] the sensitivity and NPV of POC ACR improved marginally from 0.90 to 0.91 and 0.90 to 0.98 respectively in the general population versus a cohort with a history of type 2 diabetes mellitus. McTaggart et al. [15] found improved sensitivity and NPV: 0.83 to 0.91 and 0.95 to 0.98 respectively in the general population compared to participants with hypertension. The study by Nah et al. [21] was conducted in prediabetic and diabetic populations with sensitivity of 0.92 and 0.80, and NPV of 0.99 and 0.91 respectively. Our sensitivity of 0.79 and NPV of 0.97 are in keeping with random urine samples in the general population. Although the loss of specificity (increased false positives) seen in those with diabetes mellitus in our study suggests the need for confirmatory laboratory testing, the increased sensitivity in this group of participants is valuable in detecting diabetic nephropathy. Although increased sensitivity was seen in subgroup analysis, the low PPV and high NPV remained in all sub-populations suggesting that laboratory confirmation of positive results is required while negative results exclude disease.

When using POC ACR, 82% of samples were correctly classified into the correct KDIGO albuminuria stages, however, the poor performance in those with albuminuria according to POC ACR suggests category shifting in this group. Despite POC ACR having 100% sensitivity in those with significant albuminuria (> 300 mg/g or stage A3), 50% of positive samples were false positive results. The high number of false positives and relatively few false negatives suggests that POC ACR tends to shift patients into higher concentrations of albuminuria, thus higher ACR categories. As such, negative results carry more significance, further emphasized by the 97% accuracy seen in those with POC ACR < 30 mg/g.

Surprisingly, our study showed a high prevalence of abnormal urine findings besides ACR- highlighting the extent of abnormalities which can be picked up with dipstick analysis in our population. Similar findings were demonstrated during phase one of the ARK study (haematuria: 32.6% and leukocyturia: 30.3%), while the effects of freezing at -80 °C were shown to have minimal effects (Supplementary). Our aims were not to validate these findings but rather to expose the extent of abnormalities seen. The epidemiology of CKD in LMICs is complex and often comprises the influence of multiple factors including NCDs, IDs, host and environmental factors [31]. Our findings suggest that strategies to decrease the impact of CKD should involve screening, diagnosing and managing CKD, but also screening for, and managing other risk factors [32].

We found a high prevalence of haematuria on dipstick analysis. Potential explanations for isolated haematuria could be endemic urogenital schistosmiasis, a known cause of CKD in LMICs [31], or glomerular pathology that could include infectious (or other) glomerulonephritides, urogenital tuberculosis, and interstitial nephritis. The high level of haematuria observed in the population is concerning: haematuria is associated with an increased risk of progression to ESRD [33], and plays a pathological role in renal tubular damage [34]. These dipstick findings need to be validated in our population, however previous studies have recommended dipstick analysis when screening for haematuria due to high sensitivity and reduced costs [35–38].

All the dipstick parameters except for leucocytes occurred with higher frequency in those with laboratory albuminuria; of these haematuria and glycosuria had statistical significance. This is in keeping with the role haematuria plays in various renal pathologies as well as the risk for kidney disease which is inferred from diabetes mellitus (a possible cause for glycosuria amongst others). Although not statistically significant the relatively higher prevalence of leucocytes in urine of those with laboratory negative findings was an unexpected finding. Previous studies [39–42] including a study performed on South African participants with CKD [43] showed that the presence of JC viruria was protective of developing CKD, with 43-fold higher odds of developing kidney disease in those without JC viruria. Whether these protective virurias stimulate leucocyte shedding in the urine of individuals is yet to be studied but could be a possible explanation for the raised leucocytes seen in laboratory negative individuals.

The limitations of dipstick screening such as false positives, interferences from various drugs, a lack a specificity and the need for confirmatory laboratory or microscopy testing are well known [44]. In RLS where access to central laboratory services is often limited the goals and uses of POC testing are very different to resource replete settings. Better patient outcomes with POC testing can be achieved even with decreased performance compared to laboratory-based testing if POC testing ensures improved linkage to care and retention in treatment [45]. The ability of dipstick testing to detect the potential presence of multiple risk factors for CKD and other co-morbid conditions such as diabetes mellitus, schistosomiasis, urinary tract infections, urological abnormalities, liver disease, and numerous others; together with conventional (albuminuria) and non-conventional (haematuria) markers of CKD in a single test is beneficial. Semi-quantitative (and semi-automated) results are preferable over subjective visual dipstick inspection, such semi-quantitative results aid primary care nurses who are often responsible for following screening guidelines in RLS.

Our study had several limitations: the POC testing was performed in a laboratory environment on the same day as laboratory measurements and not at the patient side, in a less controlled environment it is possible the POC instrument will not perform to the same standards. The urine samples were kept in a fridge overnight and then frozen the next day at -80 °C before being shipped frozen to the central laboratory. Previous studies have shown good stability of urine albumin and ACR with long term storage at -70 °C [46, 47], urine albumin and ACR is stable for up to 7 days when stored at 4 °C [48, 49]. To our knowledge no studies exist on the stability of urine dipstick analysis in previously frozen samples. Dipstick analysis on samples post refrigeration up to 48 h has shown: glucose to be stable up to 48 h at 4 °C; nitrites stable up to 24 h at 4 °C; leucocytes, haemoglobin, red blood cells, bilirubin show increased false negative results at 4 °C from between 4 and 8 h [50, 51]. It is possible the POC device would perform better on fresh urine as this is the recommended sample from the manufacturer (due to bilirubin and urobilinogen deterioration), however frozen and refrigerated samples can be used if returned to room temperature [52]. Despite the widespread use of immunoassays and the kinetic Jaffe method to measure laboratory ACR, these methods remain far from ideal gold standards with which to compare POC ACR. There is currently no reference method or material for urine albumin; as such methods are not standardised despite this being a goal of the National Kidney Disease Education Program [53, 54]. Comparison of results between different methods have generally not found large biases [53, 55]. Although isotope dilution mass spectrometry-traceability has been established for the Jaffe method and a primary reference material exists the lack of matrix-specific secondary reference materials for urine creatinine mean that calibration is not ideal [53]. Consequently, the use of laboratory ACR as the gold standard in our study was a pragmatic rather than ideal choice. Our study also has several advantages, the large sample size from a rural population in South Africa represents an important demographic group where POC testing may be most beneficial. While random urine sampling is not the recommended sample type (versus concentrated early morning sampling) it represents the situation that will be encountered across rural clinics in South Africa. We were able to process all 700 samples, without having to exclude outliers- and so better represent the clinical situation which would be encountered by health care workers.

## Conclusions

We have demonstrated the good rule out utility of a POC ACR dipstick device, which could aid in excluding at risk patients who do not require confirmatory central laboratory testing in RLS. We have also shown the large number of other abnormal findings seen with urine dipstick testing in our population- which could have implications for establishing screening guidelines for multiple diseases or risks for CKD with a single test. Our study population and findings are relevant in rural Africa with limited access to central laboratory services.

## Supplementary Information

**Additional file 1.** Table S1: Classification of haematuria and leukocyturia pre and post freezing compared to laboratory.

**Additional file 2.** Phase 1: FW questionnaire CKD risk – family.

**Additional file 3.** Phase 1: FW questionnaire CKD Risk - participant a.

**Additional file 4.** Phase 1: FW questionnaire CKD Risk - participant b.

## Data Availability

The data underlying this article will be shared on reasonable request to the corresponding author.

## Abbreviations

CKD: Chronic kidney disease
POC: Point-of-care
ACR: Albumin-creatinine ratio
PPV: Positive predictive value
NPV: Negative predictive value
SSA: Sub-Saharan Africa
ID: Infectious diseases
NCD: Non-communicable diseases
LMIC: Low- and middle-income countries
RLS: Resource-limited settings
SA: South Africa
ARK: African Research on Kidney
BP: Blood pressure
