# Supplementary material for: Diagnostic accuracy of semiquantitative point of care urine albumin to creatinine ratio and urine dipstick analysis in a primary care resource limited setting in South Africa
Source: BMC Nephrol. 2021 Mar 20;22:103. doi: 10.1186/s12882-021-02290-5 (PMC7981803; doi:10.1186/s12882-021-02290-5)
Supplement: Supplementary file 1 — Additional file 1. Table S1: Classification of haematuria and leukocyturia pre and post freezing compared to laboratory. [file 12882_2021_2290_MOESM1_ESM.docx]

**Supplementary Material**

**Freezing and dipstick parameters**

Methodology

The effects of sample freezing on dipstick analysis was investigated by comparing 45 samples for haematuria and leukocyturia according to the semi-quantitative POC device before and after storage at -80^0^C for seven days, in both cases this was compared to laboratory results (Beckman Coulter Iris-IQ200®) before freezing. Samples were classified as positive or negative. POC methods were considered positive if trace or above (1+, 2+, or 3+) was detected by semi-quantitative results. Laboratory considered positive for haematuria if erythrocytes >10 cells/uL and positive for leukocyturia if leukocytes > 25 cells/uL (based on cut-offs used on the POC device).

Results

Sensitivity and specificity were compared for haematuria and leukocyturia on fresh and frozen samples compared to laboratory measurements. Sensitivity for haematuria and leukocyturia decreased slightly post freezing: 78% vs 74%, and 88% vs 75% respectively. Specificity was unchanged post freezing for haematuria (68%) and slightly improved: 52% vs 67% for leukocyturia.

| **Table S1: Classification of haematuria and leukocyturia pre and post freezing compared to laboratory** | | | | |
| --- | --- | --- | --- | --- |
| **Haematuria (fresh urine)** |  |  |  |  |
|  | Positive POC (n=25) | Negative POC (n=20) |  |  |
| Positive lab (n=23) | 18 | 5 | Sensitivity | 0,78 |
| Negative lab (n=22) | 7 | 15 | Specificity | 0,68 |
|  |  |  |  |  |
| **Haematuria (post freezing)** |  |  |  |  |
|  | Positive POC (n=24) | Negative POC (n=21) |  |  |
| Positive lab ACR (n=23) | 17 | 6 | Sensitivity | 0,74 |
| Negative lab ACR (n=22) | 7 | 15 | Specificity | 0,68 |
|  |  |  |  |  |
| **Leukocyturia (fresh urine)** |  |  |  |  |
|  | Positive POC (n=31) | Negative POC (n=14) |  |  |
| Positive lab (n=24) | 21 | 3 | Sensitivity | 0,88 |
| Negative lab (n=21) | 10 | 11 | Specificity | 0,52 |
|  |  |  |  |  |
| **Leukocyturia (post freezing)** |  |  |  |  |
|  | Positive POC (n=25) | Negative POC (n=20) |  |  |
| Positive lab (n=24) | 18 | 6 | Sensitivity | 0,75 |
| Negative lab (n=21) | 7 | 14 | Specificity | 0,67 |
|  |  |  |  |  |
| Haematuria positive if red blood cells or haemoglobin detected in urine according to POC dipstick results | | | | |
