# Supplementary material for: Diagnostic accuracy of semiquantitative point of care urine albumin to creatinine ratio and urine dipstick analysis in a primary care resource limited setting in South Africa
Source: BMC Nephrol. 2021 Mar 20;22:103. doi: 10.1186/s12882-021-02290-5 (PMC7981803; doi:10.1186/s12882-021-02290-5)
Supplement: Supplementary file 2 — Additional file 2. Phase 1: FW questionnaire CKD risk – family. [file 12882_2021_2290_MOESM2_ESM.pdf]

Phase 1: FW questionnaire CKD risk - family

Agincourt HDSS study number	__________________________________
Date	__________________________________
Time	__________________________________
Have you heard of high blood pressure/hypertension/hi-hi?<br>"Xana mi tshame mi twa hi ntlakuko wa ngati/hypertension/hi hi?"	◯ no<br>◯ yes<br>◯ dont know
Have you heard of chronic kidney disease/kidney failure?<br>" Xana mi tshama mi twa hi vuvabyi bya tinso lebyi nga tshungulekiki/ ku tsandzeka ku tirha ka tinso?	◯ no<br>◯ yes<br>◯ dont know

Has any family member (your parents, your brothers/sisters, or any of your children) ever had or do they currently have any of the following diseases?

Diabetes or high sugar<br>"Mavabyi ya chukele kumbe chukele leri nga henhla"	◯ no<br>◯ yes<br>◯ dont know
Who?<br>"Kutani hi wihi?"	☐ parent/s<br>☐ brother/s and/or sisters<br>☐ child/children<br>☐ other<br>☐ dont know
Hypertension or high blood pressure<br>"Hypertension kumbe ntlakuko wa ngati"	◯ no<br>◯ yes<br>◯ don't know
Who?<br>"Kutani hi wihi?"	☐ parent/s<br>☐ brother/s and/or sisters<br>☐ child/children<br>☐ other<br>☐ dont know
Heart disease<br>"Vuvabyi bya mbilu"	◯ no<br>◯ yes<br>◯ dont know
Who?<br>"Kutani hi wihi?"	☐ parent/s<br>☐ brother/s and/or sisters<br>☐ child/children<br>☐ other<br>☐ dont know

Chest pain or angina<br>"Ku vava ka xifuva kumbe angina"	◯ no<br>◯ yes<br>◯ dont know
Who?<br>"Kutani hi wihi?"	☐ parent/s<br>☐ brother/s and/or sisters<br>☐ child/children<br>☐ other<br>☐ dont know
High cholesterol<br>"Mafurha yo tala"	◯ no<br>◯ yes<br>◯ dont know
Who?<br>"Kutani hi wihi?"	☐ parent/s<br>☐ brother/s and/or sisters<br>☐ child/children<br>☐ other<br>☐ dont know
Chronic kidney disease or kidney failure<br>"Vuvabyi bya tinso lebyi nga tshungulekiki /ku tsandzeka ku tirha ka tinso"	◯ no<br>◯ yes<br>◯ dont know
Who?<br>"Kutani hi wihi?"	☐ parent/s<br>☐ brother/s and/or sisters<br>☐ child/children<br>☐ other<br>☐ dont know
Stroke<br>"Xiome"	◯ no<br>◯ yes<br>◯ dont know
Who?<br>"Kutani hi wihi?"	☐ parent/s<br>☐ brother/s and/or sisters<br>☐ child/children<br>☐ other<br>☐ dont know
Completed by	______________________________
