# Supplementary material for: Diagnostic accuracy of semiquantitative point of care urine albumin to creatinine ratio and urine dipstick analysis in a primary care resource limited setting in South Africa
Source: BMC Nephrol. 2021 Mar 20;22:103. doi: 10.1186/s12882-021-02290-5 (PMC7981803; doi:10.1186/s12882-021-02290-5)
Supplement: Supplementary file 3 — Additional file 3. Phase 1: FW questionnaire CKD Risk - participant a. [file 12882_2021_2290_MOESM3_ESM.pdf]

Phase 1: FW questionnaire CKD Risk - participant a

Agincourt HDSS study number ______________________

Screening the participant for hypertension

Has a doctor or other health care worker ever measured your blood pressure?
"Xana dokodela kumbe mutirhi un'wana wa swarihanyo u tshame a pima ntlakuko wa ngati ya n'wina?"
- ◯ no
- ◯ yes
- ◯ dont know

Has a doctor or health worker ever told you that you have raised blood pressure or hypertension?
"Xana dokodela kumbe mtirhi un'wana wa swarihanyo u tshame a mi byela leswaku mi na ntalukuko wa ngati kumbe hypertension?"
- ◯ no
- ◯ yes
- ◯ dont know

How long have you had raised blood pressure?
"I nkarhi wo tani hi kwihi mi ri na ntlakuko wa ngati?"
- ◯ less than 1 year "hansi ka lembe rin'we"
- ◯ 1 to 5 years "lembe ri'nwe ku fika ntlhanu wa melembe"
- ◯ 6 to 10 years "tsevu wa malembe ku fika khume ra malembe"
- ◯ longer than 10 years "ku tlula khume ra malembe"

Are you currently taking medicine for high blood pressure or hypertension?
"Mi ngava mi ri ku tekeni ka vutshunguri bya ntlaluko wa ngati kumbe hypertension eka nkarhi wa sweswi?"
- ◯ no
- ◯ yes
- ◯ dont know

Have you taken your blood pressure medicine today?
"Xana mi ngava mi tekile vutshunguri bya n'wina bya ntlakuko wa ngati namuntlha?"
- ◯ no
- ◯ yes
- ◯ dont know

Screening the participant for diabetes

Has a doctor or other health care worker ever tested your blood for high sugar?
"Xana dokodela kumbe mutirhi un'wana wa swarihanyo u tshame a kambela ngati ya n'wina ku kuma loko chukele ri ngava ri tlakukile?"
- ◯ no
- ◯ yes
- ◯ dont know

Has a doctor or health worker ever told you that you have high blood sugar or diabetes?
"Xana dokodela kumbe mtirhi un'wana wa swarihanyo u tshame a mi byela leswaku mi na chukele leri tlakukeke engatini kumbe vuvabyi bya chukele?"
- ◯ no
- ◯ yes
- ◯ dont know

How long have you had raised blood sugar or diabetes?
"I nkarhi wo tani hi kwihi mi ri na chukele engatini kumbe vuvabyi bya chukele?"
- ◯ less than 1 year "hansi ka lembe rin'we"
- ◯ 1 to 5 years "lembe ri'nwe ku fika ntlhanu wa melembe"
- ◯ 6 to 10 years "tsevu wa malembe ku fika khume ra malembe"
- ◯ longer than 10 years "ku tlula khume ra malembe"

Are you currently taking medicine for high blood sugar or diabetes?<br>"Mi ngava mi ri ku tekeni ka vutshunguri bya chukele leri tlakukeke engatini kumbe bya vuvabyi bya chukele eka nkarhi wa sweswi?"	◯ no<br>◯ yes<br>◯ dont know
Have you taken your medicine for high blood sugar or diabetes today?<br>"Xana mi ngava mi tekile vutshunguri bya n'wina bya chukele leri tlakukeke engatini kumbe bya vuvabyi bya chukele namuntlha?"	◯ no<br>◯ yes<br>◯ dont know

Screening the participant for high cholesterol

Has a doctor or other health care worker ever tested your blood for high cholesterol?<br>"Xana dokodela kumbe mutirhi un'wana wa swarihanyo u tshame a mi kambela ntlakuko wa mafurha engatini ya n'wina?"	◯ no<br>◯ yes<br>◯ dont know
Has a doctor or health worker ever told you that you have high blood cholesterol?<br>"Xana dokodela kumbe mtirhi un'wana wa swarihanyo u tshame a mi byela leswaku mi na mafurha yo tala enngatini?"	◯ no<br>◯ yes<br>◯ dont know
How long have you had raised blood cholesterol?<br>"I nkarhi wo tani hi kwihi mi ri na ntlakuko wa mafurha engatini?"	◯ less than 1 year "hansi ka lembe rin'we"<br>◯ 1 to 5 years "lembe ri'nwe ku fika ntlhanu wa melembe"<br>◯ 6 to 10 years "tsevu wa malembe ku fika khume ra malembe"<br>◯ longer than 10 years "ku tlula khume ra malembe"
Are you currently taking medicine for high blood cholesterol?<br>"Mi ngava mi ri ku tekeni ka vutshunguri bya ntlakuko wa mafurha engatini eka nkarhi wa sweswi?"	◯ no<br>◯ yes<br>◯ dont know
Have you taken your medicine for high blood cholesterol today?<br>"Xana mi ngava mi tekile vutshunguri bya n'wina bya ntlakuko wa mafurha namuntlha?"	◯ no<br>◯ yes<br>◯ dont know

Screening the participant for kidney disease

Has a doctor or other health care worker ever tested your blood for kidney disease?<br>"Xana dokodela kumbe mutirhi un'wana wa swarihanyo u tshame a mi kambela vuvabyi bya tinso engatini ya n'wina?"	◯ no<br>◯ yes<br>◯ dont know
Has a doctor or health worker ever told you that you have kidney disease?<br>"Xana dokodela kumbe mtirhi un'wana wa swarihanyo u tshame a mi byela leswaku mi na vuvabyi bya tinso?"	◯ no<br>◯ yes<br>◯ dont know

How long have you had kidney disease?<br>"I nkarhi wo tani hi kwihi mi ri na vuvabyi bya tinso?"	◯ less than 1 year "hansi ka lembe rin'we"<br>◯ 1 to 5 years "lembe ri'nwe ku fika ntlhanu wa melembe"<br>◯ 6 to 10 years "tsevu wa malembe ku fika khume ra malembe"<br>◯ longer than 10 years "ku tlula khume ra malembe"
Are you currently taking medicine for kidney disease?<br>"Xana mi ngava mi teka vutshunguri bya vuvabyi bya tinso eka nkarhi wa sweswi?"	◯ no<br>◯ yes<br>◯ dont know
Have you taken your medicine for kidney disease today?<br>"Xana mi ngava mi tekile vutshunguri bya n'wina bya vuvabyi bya tinso namuntlha?"	◯ no<br>◯ yes<br>◯ dont know

Infections in the bladder or kidneys can cause any of the following symptoms:
burning when you pass urine;
the urge to pass urine frequently;
blood in the urine;
fever;
back pain;
pain in the abdomen or pelvic area

"Ku tluleriwa hi mavabyi eka buladara kumbe tinso swi nga vanga swikombiso swihi kumbe swihi swa leswi landzelaka: ku twa ku hisa/vava loko mi tsakamisa, ku va mi twa mi lava ku tsakamisa nkarhi na nkarhi, ngati eka mitsakamiso, ku twa ku hisa kumbe ku titimela, ku vava enhlanini, ku vava amasengeni kumbe eka pelvic area"

Have you ever experienced this or been told by a doctor or other health care worker that you have an infection in your bladder or kidneys?<br>"Xana mi tshame mi tokota swilo leswi kumbe ku byeriwa hi dokodela kumbe un'wana wa swarihanyo leswaku mi tluleriwile hi mavabyi eka buladara kumbe tinso n'wina?"	◯ no<br>◯ yes<br>◯ dont know
How old were you when this happened?<br>"Xana a mi ri na malembe mangani loko swilo leswi swi humelela?"	◯ younger than 18 years old "ehansi ka malembe ya khume-nhungu"<br>◯ 18 years or older "Khume-nhungu wa malembe kumbe ku ya ehenhla"<br>◯ dont know "a ndzi swi tivi"
How many times has this happened? "Xana leswi swi ngava swi humelele kangani?"<br>Once = 0, "kan'we" More than once = 1 "ku tlula kan'we"	◯ once<br>◯ more than once

Screening the participant for kidney stones

Have you ever been told by a doctor or other health care worker that you have a stone/s in your kidney? "Xana mi tshame my byeriwa hi dokodela kumbe mutirhi un'wana wa swarihanyo leswaku mi na ribye/maribye eka yinso ya n'wina?"	◯ no<br>◯ yes<br>◯ dont know
How old were you when this happened? "Xana a mi ri na malembe mangani loko swilo leswi swi humelela?"	◯ younger than 18 years old "ehansi ka malembe ya khume-nhungu"<br>◯ 18 years or older "Khume-nhungu wa malembe kumbe ku ya ehenhla"<br>◯ dont know "a ndzi swi tivi"
How many times has this happened? "Xana leswi swi ngava swi humelele kangani?" Once = 0, "kan'we" More than once = 1 "ku tlula kan'we"	◯ once<br>◯ more than once
Has any family member ever been told by a doctor or other health care worker that they have kidney stones? "Xana xirho xihi kumbe xihi xa ndyangu xi ngava xi tshame xi byeriwa hi dokodela kumbe mutirhi wa swarihanyo leswaku xi na swiribyana eka tinso?"	◯ no<br>◯ yes<br>◯ dont know
Who? "Mani?"	☐ parent/s<br>☐ brother/s and/or sisters<br>☐ child/children<br>☐ other<br>☐ dont know

Screening the participant for urinary bilharzia

Have you ever seen blood in your urine? "Xana mi tshame mi vona ngati eka mitsakamiso ya n'wina?" (for women - if you have seen blood in your urine at the time of menstruation this does not count) ("eka vavasati - loko mi vone ngati eka mitsakamiso ya n'wina hi nkarhi lowu mi nga emasikwini a swi vuli nchumu")	◯ no<br>◯ yes<br>◯ dont know
Have you seen blood in your urine in the last 12 months? "Xana mi tshame mi vona ngati eka mitsakamiso ya n'wina eka tin'hweti ta khume-mbirhi leti nga hundza?"<br><br>(for women - if you have seen blood in your urine at the time of menstruation this does not count) ("eka vavasati - loko mi vone ngati eka mitsakamiso ya n'wina hi nkarhi lowu mi nga emasikwini a swi vuli nchumu")	◯ no<br>◯ yes<br>◯ dont know
Have you ever swum in the rivers in the area, collected water from the river or washed clothes in the river? "Xana mi tshame mi hlambela eminkoveni laha ndzhawini, kumbe ku ka mati enkoveni kumbe ku hlantswa swimbalo enkoveni?"	◯ no<br>◯ yes<br>◯ dont know

Has a doctor or other health care worker ever tested your urine for bilharzia?<br>"Xana dokodela kumbe mutirhi un'wana wa swarihanyo u tshame a kambela bilharzia eka mitsakamiso ya n'wina?"	◯ no<br>◯ yes<br>◯ dont know
Has a doctor or health worker ever told you that you have bilharzia in your urine?<br>"Xana dokodela kumbe mutirhi wa swarihanyo u tshame a mi byela lewsaku mi na bilharzia eka mitsakamiso ya n'wina?"	◯ no<br>◯ yes<br>◯ dont know
Did you receive treatment?<br>"Mi ngava mi kumile vutshunguri?"	◯ no<br>◯ yes<br>◯ dont know
How many times have you received treatment?<br>"Xana mi kumile vutshunguri nkarhi wo fika kwihi?"	◯ once "kanwe"<br>◯ 2 to 5 times "kambirhi ku fika ka ntlhanu"<br>◯ more than 5 times "ku tlula ka ntlhanu"<br>◯ dont know "a ndzi swi tivi"
Completed by	__________________________
