# Supplementary material for: Diagnostic accuracy of semiquantitative point of care urine albumin to creatinine ratio and urine dipstick analysis in a primary care resource limited setting in South Africa
Source: BMC Nephrol. 2021 Mar 20;22:103. doi: 10.1186/s12882-021-02290-5 (PMC7981803; doi:10.1186/s12882-021-02290-5)
Supplement: Supplementary file 4 — Additional file 4. Phase 1: FW questionnaire CKD Risk - participant b. [file 12882_2021_2290_MOESM4_ESM.pdf]

Phase 1: FW questionnaire CKD Risk - participant b

Agincourt HDSS study number ________________________

Screening the participant for other chronic conditions (this excludes high blood pressure, diabetes, high cholesterol, kidney disease, kidney stones, urinary tract infection, urinary bilharzia and hiv because we have already asked about this)

Have you ever taken treatment for any other chronic disease?<br>"Xana mi tshame teka vutshunguri by mavabyi wahi kumbe wahi yo ka ya nga tshunguleki?"	◯ no<br>◯ yes<br>◯ dont know
For what disease was it?<br>"Xana a ku ri bya yini?"	☐ TB "vuvabyi bya rifuva"<br>☐ cancer "mfukuzana"<br>☐ other "swi'nwana"
Describe what the "other" illness is	________________________
For how long did you take the treatment?<br>"Xana mi tekile nkarhi wo fika kwihi vutshunguri lebyi?"	◯ one month "n'hweti yin'we"<br>◯ 2-6 months "tin'hweti timbirhi ku fika tsevu"<br>◯ 7-12 months "nkombo wa tin'hweti ku fika khume-mbirhi"<br>◯ more than 12 months "ku tlula khume-mbirhi wa tin'hweti"
Are you currently taking this treatment?<br>"Xana mi ngava ma ha teka vutshunguti lebyi eka nkarhi wa seswi?"	◯ no<br>◯ yes<br>◯ dont know
Are you currently taking any pain killers?<br>"Xana mi ngava mi teka tiphilisi to karhi to herisa ku vava eka nkarhi wa sweswi?"	◯ no<br>◯ yes<br>◯ dont know
How long have you been taking the pain killers?<br>"Xana mi na nkarhi wo fika kwihi mi teka tiphilisi to herisa ku vava?"	◯ one month or less "ehansi ka n'hweti yin'we "<br>◯ 2-6 months "tin'hweti timbirhi ku fika tsevu"<br>◯ 7-12 months "nkombo wa tin'hweti ku fika khume-mbirhi"<br>◯ more than 12 months "ku tlula khume-mbirhi wa tin'hweti"

Screening the participant for tobacco exposure

Have you ever smoked any tobacco product such as cigarettes, cigars or pipes?<br>"Xana mi tshame mi dzaha fole ro karhi ku fana na sigarete, cigars kumbe fole ro dzaha hi phayiphi?"	◯ no<br>◯ yes<br>◯ dont know
How old were you when you first started smoking?<br>"Xana a mi ri na malembe mangani loko mi sungula ku dzaha?"	◯ younger than 18 years old "ehansi ka malembe ya khume-nhungu"<br>◯ 18 years or older "Khume-nhungu wa malembe kumbe ku ya ehenhla"<br>◯ dont know "a ndzi swi tivi"

Have you quit smoking?<br>"Xana mi ngava mi tshikile ku dzaha?"	◯ no<br>◯ yes<br>◯ dont know
How often do you smoke tobacco products?<br>"Xana mi ngava mi dzaha kangani fole?"	◯ every day "siku rin'wana na rin'wana"<br>◯ 5 to 6 days per week "ntlanu ku fika tsevu wa masiku evhikini"<br>◯ 1 to 4 days per week "siku ku fika mune wa masiku evhikini"<br>◯ 1 to 3 days per month "siku ku fika masiku manharhu en'hwetini"<br>◯ less than once per month "ehansi ka nkarhi wun'we en'hwetini"<br>◯ when i get a chance "loko ndzi kuma nkarhi"
Do you currently inhale any tobacco products (e.g. snuff)?<br>"Xana mi ngava mi dzaha muxaka wun'wana wa fole eka nkarhi wa sweswi (xikombiso xinefu)?"	◯ no<br>◯ yes<br>◯ dont know
How frequently?<br>"Mi dzaha kangani?"	◯ daily "siku rin'wana na rin'wana"<br>◯ 2-3 x per week "masiku mambirhi ku fika manharhu evhikini"<br>◯ once a week "kan'we hi vhiki"<br>◯ once a month "kan'we hi n'hweti"<br>◯ when i get a chance "loko ndzi kuma nkarhi"

Screening the participant for traditional medicine usage

Have you ever taken traditional medicine?<br>"Xana mi tshame mi teka vutshunguri bya xinto?"	◯ no<br>◯ yes<br>◯ dont know
How many times have you taken traditional medicine in the last 12 months?<br>"Xana mi tekile kangani vutshunguri bya xinto eka tin'hweti ta khume-mbirhi leti nga hundza?"	◯ once "kan'we"<br>◯ twice "kambirhi"<br>◯ three times "kanharhu"<br>◯ more than three times "ku tlula kanharhu"<br>◯ not in the last 12 months "kungari eka tin'hweti ta khume-mbirhi leti nga hundza"
How did you take the traditional medicine?<br>"Xana mi tekile njhani vutshunguri lebyi bya xintu?"	☐ orally "hi ku nwa hi nomo"<br>☐ enema "hi ku va mi chuluka"<br>☐ other "swin'wana"

Screening female participants for obstetric risk

Have you ever been pregnant?<br>"Xana mi tshame mi biha emirini?"	◯ no<br>◯ yes<br>◯ dont know
How pregnancies have you had?<br>"Xana mi bihile emirini kangani?"	◯ one "kan'we"<br>◯ two "kambirhi"<br>◯ three "kanharhu"<br>◯ more than three "ku tlula kanharhu"

Did you have high blood pressure during any of your pregnancies?<br>"Xana mi vile na ntlakuko wa ngati nkarhi wa vuyimani byihi kumbe byihi bya n'wina?"	◯ no<br>◯ yes<br>◯ dont know
Did the high blood pressure result in any one or more of the following: premature delivery, stillbirth, abortion<br>" Xana ntlakuko wa ngati wu ngava wu vangile xin'we kumbe swo tala swa leswi landzelaka: ku bebula nkarhi wu nga se fika, ku bebula n'wana la loveke, ku huma ka khwirhi?"	◯ no<br>◯ yes<br>◯ dont know
Are you using any contraception?<br>"Xana mi tirhisa nkunguhato wo karhi?"	◯ no<br>◯ yes<br>◯ dont know
What type?<br>"I muxaka muni wa nkunguhato?"	◯ oral contraceptive "nkunguhato wo nwa"<br>◯ intrauterine device "nkunguhato lowu vekeriwaka eka xivelekelo"<br>◯ injectable "ku tlhava"<br>◯ sterilization "ku susa xivelekelo"<br>◯ barrier methods "maendlelo man'wana yo sivela ku bebula"<br>◯ other "swinwana"
Completed by	______________________________
